# An Essential Postdevelopmental Role for Lis1 in Mice

**DOI:** 10.1523/ENEURO.0350-17.2018

**Published:** 2018-02-02

**Authors:** Timothy J. Hines, Xu Gao, Subhshri Sahu, Meghann M. Lange, Jill R. Turner, Jeffery L. Twiss, Deanna S. Smith

**Affiliations:** 1 Department of Biological Sciences, University of South Carolina, Columbia, SC 29208; 2 Quantshine Asset Mgmt. Co. Ltd, Shanghai 200082, China; 3 College of Pharmacy Drug Discovery and Biomedical Sciences, University of South Carolina, Columbia, SC 29208

**Keywords:** axonal transport, brainstem, cytoplasmic dynein, knockout mouse, Lis1, neurological disease

## Abstract

*LIS1* mutations cause lissencephaly (LIS), a severe developmental brain malformation. Much less is known about its role in the mature nervous system. LIS1 regulates the microtubule motor cytoplasmic dynein 1 (dynein), and as LIS1 and dynein are both expressed in the adult nervous system, Lis1 could potentially regulate dynein-dependent processes such as axonal transport. We therefore knocked out Lis1 in adult mice using tamoxifen-induced, Cre-ER-mediated recombination. When an actin promoter was used to drive Cre-ER expression (Act-Cre-ER), heterozygous Lis1 knockout (KO) caused no obvious change in viability or behavior, despite evidence of widespread recombination by a Cre reporter three weeks after tamoxifen exposure. In contrast, homozygous Lis1 KO caused the rapid onset of neurological symptoms in both male and female mice. One tamoxifen-dosing regimen caused prominent recombination in the midbrain/hindbrain, PNS, and cardiac/skeletal muscle within a week; these mice developed severe symptoms in that time frame and were killed. A different tamoxifen regimen resulted in delayed recombination in midbrain/hindbrain, but not in other tissues, and also delayed the onset of symptoms. This indicates that Lis1 loss in the midbrain/hindbrain causes the severe phenotype. In support of this, brainstem regions known to house cardiorespiratory centers showed signs of axonal dysfunction in KO animals. Transport defects, neurofilament (NF) alterations, and varicosities were observed in axons in cultured DRG neurons from KO animals. Because no symptoms were observed when a cardiac specific Cre-ER promoter was used, we propose a vital role for Lis1 in autonomic neurons and implicate defective axonal transport in the KO phenotype.

## Visual Abstract

**Figure:**
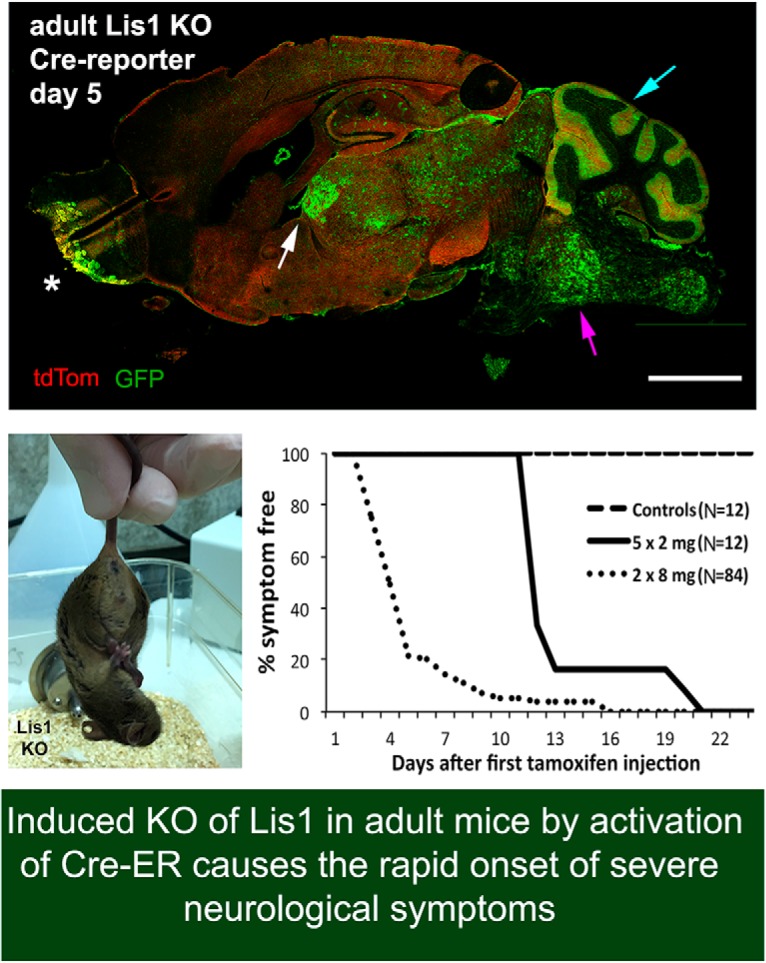

## Significance Statement

Mammalian Lis1 is best known for its role in brain development. Lis1 binds to and regulates the microtubule motor, cytoplasmic dynein. We show that Lis1 function is needed post-developmentally and provide evidence that loss of Lis1 in the hindbrain leads to death. The effect is dose dependent in mice, as loss of only one allele does not produce an overt phenotype. However, since LIS1 haploinsufficiency causes lissencephaly (LIS) in humans, our study raises the possibility that post-developmental axonal transport defects could contribute to worsening symptoms in children with LIS1 mutations. Our data are consistent with the hypothesis that Lis1 regulates dynein-dependent axon transport in the mature nervous system.

## Introduction

*LIS1* mutations in humans cause a “smooth brain” malformation called lissencephaly (LIS) characterized by severe cognitive and motor impairments and worsening epilepsy, leading to early mortality (Dobyns et al., 1993; Sapir et al., 1999; Gleeson, 2000; Sicca et al., 2003; Saillour et al., 2009; Reiner and Sapir, 2013; Dobyns and Das, 2014; Herbst et al., 2016). Most of the human mutations result in a null allele with ∼50% reduction of LIS1 protein levels, which profoundly impacts the developing nervous system. Other mutations can produce a milder phenotype, but the phenotype/genotype correlation is complex. A classic mouse study made it clear that gene dosage is relevant, as progressive reduction of Lis1 protein levels caused progressively more severe phenotypes (Hirotsune et al., 1998). Deletion of a large portion of one Lis1 allele in mice, resulting in a null allele, delays neuronal migration and differentiation, but unlike humans, mature mice show mild neurologic abnormalities and are viable and fertile (Hirotsune et al., 1998; Gambello et al., 2003). Cre-mediated knockout (KO) in specific subpopulations of developing neural cells in mice impacts mitosis and nucleokinesis, causing developmental delay (Tsai et al., 2005; Tsai et al., 2007; Yingling et al., 2008; Youn et al., 2009; Hippenmeyer et al., 2010).

Lis1 is a highly conserved regulator of the minus-end directed microtubule motor protein, cytoplasmic dynein 1; together they regulate neural stem cell spindle orientation, nucleokinesis, and nuclear envelope breakdown during brain development (Vallee et al., 2001; Wynshaw-Boris and Gambello, 2001; Gambello et al., 2003; Shu et al., 2004; Tsai et al., 2005, 2007; Vallee and Tsai, 2006; Hebbar et al., 2008; Schwamborn and Knoblich, 2008; Yingling et al., 2008; Youn et al., 2009; Hippenmeyer et al., 2010; Moon et al., 2014). In fact, mutations in the dynein heavy chain gene *DYNC1H1* can also cause cortical malformations in humans (Vissers et al., 2010; Willemsen et al., 2012; Poirier et al., 2013).

Of particular interest are reports that DYNC1H1 mutations cause later onset neurologic disorders, including forms of spinal muscular atrophy (SMA) and Charcot-Marie-Tooth disease (Weedon et al., 2011; Harms et al., 2012). Additionally, mutations in genes encoding two other dynein regulators DCTN1 and BICD2, cause Perry syndrome and SMA (Rees et al., 1976; Wider and Wszolek, 2008; Neveling et al., 2013; Oates et al., 2013; Peeters et al., 2013). The extent to which Lis1 functions post-developmentally, especially in minimally proliferative tissues like adult brain, has not been studied extensively. Hunt et al. (2012) found that heterozygous *Lis1* KO in six-week-old mice altered synaptic function in the hippocampus in the absence of altered laminar granule cell architecture, but the mechanisms underlying the altered activity are not known (Hunt et al., 2012). It has been shown that Lis1 manipulations impact dynein-dependent axon transport in sensory neurons cultured from adult rats (Smith et al., 2000; Pandey and Smith, 2011). These results suggest that Lis1 is a positive regulator of dynein-based axon transport in adult mammals. This was also found in adult mouse DRG neurons (Klinman and Holzbaur, 2015). Although axon transport studies suggest a role for Lis1 in cultured adult neurons, these neurons do not form synaptic connections, so its involvement in synapse formation and maturation is currently unknown. If Lis1 indeed regulates axon transport in the mature nervous system, Lis1 mutations could have deleterious effects on circuitry in mature brains. We have addressed this fundamental question using a tamoxifen-inducible Cre-Lox system to disrupt Lis1 selectively in adult mice. We show that Lis1 is indispensable in adult mice, and describe unexpected temporal and spatial recombination patterns and how they impact the phenotype of Lis1 KO in adult animals. Our data point to a vital role for Lis1 in cardiorespiratory nuclei in the hindbrain.

## Materials and Methods

### Mice

All animal experiments were conducted under a protocol approved by the Animal Care and Use Committee of the University of South Carolina. Males and females were used in experiments; no differences were observed in outcomes between males and females. Four mouse strains were used to generate the inducible Lis1 KO mice (Table 1). (1) *129S-Pafah1b1^tm2Awb^/J* (The Jackson Laboratory 008002, RRID:IMSR_JAX:008002): *loxP* sites flank exons 3–6. Homozygous mice are viable and fertile, but have mild hippocampal abnormalities and express ∼75% of WT Lis1 levels (Hirotsune et al., 1998); (2) Tg(CAG-cre/Esr1*)5Amc/J (The Jackson Laboratory 004453, RRID:IMSR_JAX:004453), a chicken β-actin promotor drives expression of Cre recombinase fused to a modified estrogen receptor; (3) Tg(Myh6-cre/Esr1*)1Jmk/J (The Jackson Laboratory 005650, RRID:IMSR_JAX:005650), expression of Cre-ER is under the control of a cardiac-specific α-myosin heavy chain promoter so that tamoxifen stimulates recombination only in cardiac cells; and (4) *Gt(ROSA)26Sor^tm4(ACTB-tdTomato,-EGFP)Luo^/J* (The Jackson Laboratory 007576, RRID:IMSR_JAX:007576), a Cre reporter mouse with *loxP* sites flanking a membrane-targeted tdTomato cassette that is positioned upstream of a membrane-targeted EGFP cassette. All cells in these mice exhibit membrane-associated red fluorescence until the tdTomato cassette is deleted by Cre recombinase for expression of membrane-associated EGFP fluorescence, allowing visualization of both recombined and non-recombined cells in the same tissue (Muzumdar et al., 2007). Table 1 shows the crosses that were used to generate experimental animals, and shows the descriptive names used for each throughout the manuscript. All strains used in experiments were homozygous for the Cre reporter. Genotyping of all animals was performed using primers and protocols recommended by The Jackson Laboratory. Primers are available on request.

**Table 1.** Mouse strains used in these studies

**Founder mouse lines**	
**The Jackson Laboratory strains**	**Descriptive name used in paper**
129S-Pafah1b1^tm2Awb^/J	Lis1^LoxP/+^ or Lis1^LoxP/LoxP^
Tg(CAG-cre/Esr1*)1Jmk/J	Act-Cre-ER (heterozygous)
Tg(Myh6-cre/Esr1*) 1Jmk/J	Myh6-Cre-ER (heterozygous)
Gt(ROSA)26Sor^tm4(ACTB-tdTomato, -EGFP)Luo^/J	Cre Reporter (heterozygous)
**All strains below are homozygous for the Cre reporter**	
**Lis1 KO strains**	**Descriptive name used in paper**
Lis1^LoxP/LoxP^ x Act-Cre-ER (het)	Lis1 KO
Lis1^LoxP/LoxP^ x Myh6-Cre-ER (het)	Myh6 KO
**Control strains**	**Descriptive name used in paper**
Act-Cre-ER	No flLis1 Control
Lis1^LoxP/LoxP^	No Cre Control
Lis1^LoxP/+^ x Act-Cre-ER Lis1	KO Het

### Tamoxifen administration

Numbers of animals are provided below (Experimental design and statistical analysis). Tamoxifen was delivered by intraperitoneal or intracerebroventricular injections in adult mice (two to five months old). For intraperitoneal delivery, mice were injected with 40 mg/ml tamoxifen (Sigma-Aldrich) dissolved in 10% ethanol and 90% corn oil (Sigma). Two different daily tamoxifen dosage regimens were tested based a previous study using this Cre-ER strain (Hayashi and McMahon, 2002): regimen 1 (5 × 2 mg), 2 mg for five consecutive days (total 10 mg); and regimen 2 (2 × 8 mg**)**, 8 mg injected on two consecutive days (total 16 mg). For intracerebroventricular delivery, mice were anesthetized with an isoflurane/oxygen vapor mixture (5% induction, 2–3% maintenance) and placed in a stereotaxic device (Kopf Instruments). Five microliters of 50 mM (Z) 4-hydroxytamoxifen (4-OHT; Sigma) dissolved in 100% ethanol was infused into the left lateral ventricle at a rate of 0.4 µl/min using a 5 µl Hamilton syringe. The needle was left in place for one additional min before removal to allow for diffusion from the injection site. Coordinates (-1.0 mm posterior from bregma, ±1.0 mm mediolateral, and -2.5 mm ventral to skull surface) were determined using the atlas of Paxinos and Franklin (Franklin and Paxinos, 2001).

### Analysis of Cre-mediated recombination by tdTomato/EGFP fluorescence

Images shown in all figures are representative of data acquired from at least *N* = 3 animals per experiment. Animals under deep isoflurane anesthesia were perfused transcardially with ice-cold PBS, followed by 4% paraformaldehyde (PFA) in 0.1 M PBS (pH 7.4). Before sectioning, tissues were cryoprotected by immersion overnight in 15% sucrose, followed by 24 h in 30% sucrose in PBS. Tissues were then frozen in OCT compound (Fisher) using a beaker of 2-methylbutane chilled in dry ice. Ten- or 50-µm-thick cryosections were stored at −80°C until use. Whole brains and hearts were imaged immediately after dissection using an Olympus SZX-12 with an SZX-RFL2 coaxial fluorescence attachment. Cryosections were imaged using a Leica TCS SP8X confocal microscope equipped with LAS X software and a 63× oil immersion objective (1.4 N/A). Some images were obtained using a Zeiss Axiovert 200 inverted microscope equipped with AxioVision software and a Plan-Neo 100 Å∼/1.30 and Plan-Apo 63 Å∼/1.40 oil-immersion objectives (Immersol 518F; Carl Zeiss, Inc.) or a Plan-Neofluor 20× dry objective.

### Protein isolation and immunoblotting

**Figure 1.**
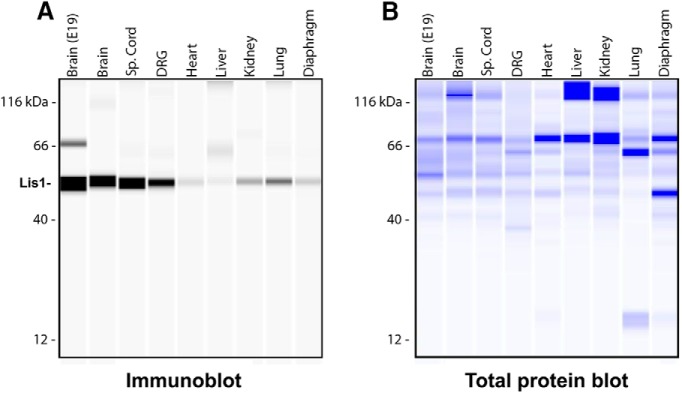
Lis1 protein is expressed in adult mouse tissues. A total of 1 µg of tissue lysates was analyzed using the Wes Simple Western System. Brain extracts from E19 were loaded as a positive control. All other extracts are from two-month-old animals. The size-based separation is processed by Compass software and displayed as virtual blots/gels. ***A***, Immune detection of Lis1 in protein samples, depicted in a virtual immunoblot generated by the system. ***B***, Total protein detection, visualized by a virtual Coomassie gel generated by the system. These blots are representative of three experimental repeats (*N* = 3).

**Figure 2.**
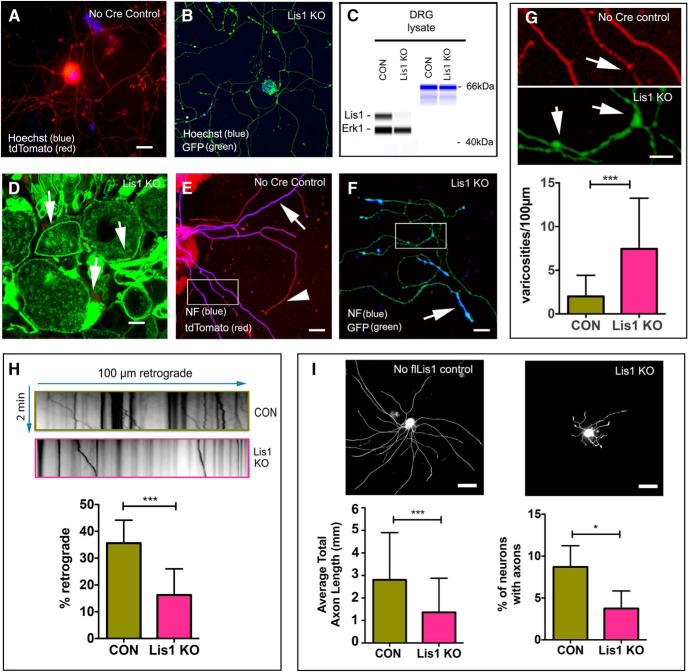
Lis1 KO impacts axonal function in adult mouse DRG neurons. ***A***, Cultured DRG neurons from no Cre control exposed to 4-OH tamoxifen for 5 d expressed only tdTomato (red) showing no signs of recombination. ***B***, In contrast, Lis1 KO neurons had strong GFP expression (green) demonstrating recombination. ***C***, 4-OH tamoxifen reduced Lis1 protein levels in Lis1 KO neurons relative to no Cre control neurons (CON). ***D***, Intraperitoneal injection of 2 × 8 mg tamoxifen in Lis1 KO mice resulted in GFP expression in intact DRGs after 4 d. Arrows point to DRG plasma membranes. ***E***, Cultured DRG neurons prepared from intraperitoneally injected, no Cre control animals expressed only tdTomato (red). NF (blue) was prominent along axon shafts (white arrow) but less prominent in axon terminals (arrowhead). ***F***, DRG neurons prepared from intraperitoneally injected Lis1 KO mice continued to express GFP (green) in culture, and NF (blue) was most prominent in distal axons and enriched in in varicosities (arrow). ***G***, Insets from ***E***, ***F*** have been digitally enlarged to show axonal varicosities (arrows). The bar graph in ***G*** shows the average number of varicosities per 100 µm of axon from *N* = 3 CON (45 mm total axon length) and three Lis1 KO (27 mm total axon length) mice. ***H***, Kymographs were generated from time-lapse movies of LysoTracker labeled organelles in GFP-positive axons. The bar graph shows the percentage moving retrogradely in Lis1 KO and no flLis1 control cultures (CON). A total of 27 100 µm axon segments were analyzed from *N* = 2 CON and *N* = 2 Lis1 KO mice. A total of *n* = 521 control and *n* = 699 KO organelles were analyzed. ***I***, Cultured DRG neurons prepared from intraperitoneally injected, no flLis1 control and Lis1 KO mice were immunostained with neuron-specific antibodies, and the percentage of neurons with growing axons was determined from *N* = 4 CON and *N* = 5 Lis1 KO mice. A total of *n* = 2219 control neurons and *n* = 2410 Lis1 KO neurons were analyzed. Bars in ***G-I*** indicate mean ± SD. Significance determined by Mann–Whitney test (***G***), Student’s *t* test (***H***, ***I***), *p < 0.05, ***p < 0.001 (see Table 2 for details). Scale bars: 20 µm (***A***, ***D***, ***E***), 5 µm (***B***), and 50 µm (***I***).

All blots are representative of at least *N* = 3 repeats. Tissues were dissected quickly from CO_2_-killed mice and frozen in liquid nitrogen, followed by Dounce homogenization in ice-cold RIPA lysis buffer with protease and phosphatase inhibitors (Thermo). Total protein in extracts was determined using a BCA assay (Thermo). Automated capillary electrophoresis and immunoblotting (Figs. 1, 2) was performed with the Wes Simple Western system using the manufacturer’s protocol (Protein Simple). One microgram of lysate was loaded for each sample. Anti-mouse (ERK1), anti-rabbit (Lis1), and total protein detection modules were used per manufacturer’s instructions. Blots were analyzed using Compass Software (Protein Simple). For traditional Western blotting (Figs. 4, 7) 10 µg of each sample were separated on 10% acrylamide gels, then transferred to PVDF membrane. Blots were probed with antibodies against Lis1 and dynein intermediate chain and proteins were detected by chemiluminescence.

### Sciatic nerve transmission electron microscopy

While anesthetized with isoflurane, WT mice were perfused with PBS, and then buffered 2.5% glutaraldehyde. Nerves were removed and fixed overnight in 2.5% glutaraldehyde, then sectioned and stained with osmium tetroxide for imaging on a JEOL 200CX Transmission Electron Microscope.

### Antibodies

Primary antibodies used are as follows: Lis1 rabbit polyclonal 484/485 (Smith et al., 2000; WB: 1:500); Lis1 rabbit polyclonal (Wes: 1:25, WB: 1:500; Santa Cruz Biotechnology sc-15319, RRID:AB_2159891); ERK1 rabbit polyclonal (Wes: 1:100; Abcam ab109282, RRID:AB_10862274); a mix of the pan-axonal neurofilament (NF) mouse monoclonal cocktail (IF: 1:500; BioLegend 837904, RRID:AB_2566782); NF light, medium, and heavy chain chicken polyclonals (IF: 1:500; Aves NFL, NFM, and NFH, RRIDs: AB_2313553, AB_2313554, AB_2313552); and the NF 200-kDa mouse monoclonal, clone RT97 (IF: 1:500; Millipore CBL212, RRID:AB_93408) were used to label NFs; GAP43 rabbit polyclonal (IF: 1:500; Novus Biologicals, NB300-143, RRID: AB_10001196); beta-III tubulin chicken polyclonal (IF: 1:500; Millipore AB9354, RRID:AB_570918); peripherin chicken polyclonal (IF: 1:500; Abcam ab39374, RRID:AB_777207); choline acetyltransferase goat polyclonal (IF: 1:100; Millipore AB144P, RRID:AB_11214092); MAP2 chicken polyclonal (IF: 1:100; Abcam ab5392, RRID:AB_2138153); myelin basic protein chicken polyclonal (IF: 1:500; Aves MBP, RRID:AB_2313550); α-tubulin mouse monoclonal (WB: 1:2000; Sigma-Aldrich T5168, RRID:AB_477579); and dynein intermediate chain mouse monoclonal (WB: 1:1000; Santa Cruz Biotechnology sc-13524, RRID:AB_668849). Secondary antibodies used: HRP-conjugated goat anti-rabbit and mouse (WB: 1:50 000; Millipore 12-348 and 12-349, RRIDs:AB_390191 and AB_390192); Cy5-conjugated donkey anti-chicken, mouse, and goat (IF: 1:250; Jackson ImmunoResearch 703-175-155, 715-175-150, and 705-175-147, RRIDs:AB_2340365, AB_2340819, and AB_2340415); and DyLight 405-conjugated donkey anti-chicken (IF: 1:250; Jackson ImmunoResearch 703-475-155, RRID: AB_2340373).

### Preparation of DRG cultures

Cultures were generated from two- to five-month-old Lis1 KO and no Cre or no flLis1 control mice. In some experiments mice were exposed to the 2 × 8 mg tamoxifen intraperitoneal regimen then neurons harvested on day 4 after the first injection. In other experiments, 4-OHT (2 µM) was added directly to the DRG cultures with these same genotypes without previous intraperitoneal injections. DRGs were harvested (∼20 per mouse), dissociated in type XI collagenase (Sigma) for 1 h at 37°C, and then triturated through a flamed Pasteur pipet. Dissociated neurons incubated in 0.05% trypsin (Invitrogen) at 37°C for 15 min. After a second trituration, cell suspensions were centrifuged through a 12.5% BSA solution to remove myelin fragments. Cells were then plated onto sterile, German glass coverslips (Fisher) coated with 10 μg/ml poly-D-lysine (>300 kDa; Sigma) and 10 μg/ml laminin (Millipore). Cells were cultured in DMEM/F12 medium (Corning) with 25 mM HEPES, GlutaMAX (Thermo Fisher), N-2 supplement (Life Technologies), and 10% horse serum (HyClone). In some experiments, 100 µM cytosine arabinoside was added to reduce non-neuronal cell proliferation.

### Axonal varicosity analysis

DRG neurons obtained after intraperitoneal injection were maintained in culture for 4 d and then fixed for 10 min in 4% PFA. Coverslips were mounted on glass slides using Prolong Gold Antifade (LifeTechnologies). Neurons were imaged using the ImageXpress XLS high content imaging system (Molecular Devices) equipped with a 20× objective. A segmented line (one-pixel width) was used to trace the axon using ImageJ software. Axonal swellings that protruded visibly beyond the one-pixel line on both sides were counted as varicosities. tdTomato-positive axons from *N* = 3 no Cre cultures (45 mm of total axon length) and EGFP-positive axons from *N* = 3 Lis1 KO cultures (27 mm of total axon length) were measured.

### Axon growth/length analysis

DRG neurons obtained after intraperitoneal injection were maintained in culture for 2 d and then fixed for 10 min in 4% PFA. Neurons were processed for immunofluorescence using a cocktail of neuron-specific antibodies: chicken anti-peripherin, chicken anti-β III tubulin, chicken anti-NF (light, medium, and heavy chains), and rabbit anti-GAP43 to ensure all axons were uniformly labeled. Neurons were imaged using the ImageXpress Micro XLS system and axon lengths were measured using WIS-Neuromath software. *N* = 5 Lis1 KO mice, and *N* = 4 no flLis1 control mice were analyzed; *n* = 122 control and *n* = 70 KO neurons were measured.

### Organelle movement

Cultured DRG neurons obtained after intraperitoneal injection were exposed to 100 nM Lysotracker-Red (Millipore Inc.) for 20 min. Coverslips were transferred into fresh medium containing OxyFluor (Oxyrase Inc.) and 25 mM HEPES (pH 7.4), and placed in a custom-built water-heated microscope stage warmed to 37°C. Organelles were imaged using a Zeiss Axiovert 200 microscope equipped with a C-Apo 63×/1.2 W/0.2 water-immersion objective. Images were acquired at 0.5-s intervals for 2 min using a Zeiss AxioCam HRm charge-coupled camera and linked AxioVision 4.7 software. Kymographs were generated from time-lapse movies using ImageJ software. *N* = 2 mice of each genotype were analyzed, with a total of 27 axon segments analyzed from each genotype. A total of *n* = 521 no flLis1 control and *n* = 699 Lis1 KO organelles were included. Direction of movement was determined by locating the cell body before imaging. Net displacement of ≥5 μm toward the cell body was categorized as retrograde.

### Immunofluorescence in DRG cultures and tissue cryosections

Immunofluorescence experiments were performed in triplicate (*N* = 3 mice per genotype) and representative images are shown in the figures. After permeabilization with 0.1% Triton X-100 for 10–30 min, samples were blocked in 3% BSA (Fisher), 10% normal goat serum (Sigma), and 0.2% Tween 20 (Bio-Rad) in PBS for 1 h. Cultures and nerve sections were exposed to primary antibodies for 1 h at room temperature. Brain and spinal cord sections were exposed to primary antibody overnight at 4°C. In both cases samples were exposed to fluorophore-conjugated secondary antibodies for 1 h at room temperature. Nuclei were stained with Hoechst dye and samples mounted using Prolong Gold.

### Quantifying chromatolysis in brainstem sections

Animals were exposed to the 2 × 8 mg tamoxifen regimen. On day 4, coronal cryosections of brainstem from *N* = 3 no flLis1 control mice (*n* = 331 neurons) and *N* = 4 Lis1 KO mice (*n* = 583 neurons) were stained with 1% toluidine blue and measured for nuclear enlargement and nuclear acentricity. The Allen Mouse Brain Atlas was used as a guide to select coronal brainstem sections in which the nucleus ambiguus and other cardiorespiratory centers were likely located. Landmarks such as the fourth ventricle and pyramus granular layers were used to identify the proper sections. Comparisons were made from matched sections. Substantial Act-Cre-ER mediated recombination (GFP expression) was consistently observed in similar sections following tamoxifen administration. ImageJ was used to determine nuclear and somal areas and centroids. The ratio of the nuclear area to the somal area was calculated to establish a “nuclear enlargement index.” A “centroid displacement index” was calculated by summing the X and Y displacement differences between the nuclear centroid and the somal centroid of each cell.

### RNA extraction and analysis

RNA analysis was performed using tissues from 3 mice per treatment and genotype. RNA was isolated from flash frozen tissues using the QIAGEN RNAEasy kit per the manufacturer’s instructions. RNA concentration was determined by fluorimetry using Ribogreen reagent (Thermo Fisher). A total of 100 ng RNA was reverse transcribed with a SensiFast cDNA Synthesis kit (Bioline). These cDNAs were used for quantitative droplet digital PCR (ddPCR) with Evagreen detection reagent and ddPCR Supermix (Bio-Rad). Droplets for ddPCR were made using a QX200 Droplet Generator (Bio-Rad). Results were analyzed using Poisson distribution on the QX200 Droplet Reader. Mouse Lis1 primer sequences (forward, 5’ GCGAACTCTCAAGGGCCATA 3’ and reverse, 5’ CATTGTGATCGTGACCGTGC 3’) were designed using NCBI BLAST (NCBI accession #NM_013625). Mouse β2 microglobulin (B2M) primers (forward, 5’ TTCTGGTGCTTGTCTCACTGA 3’ and reverse, 5’ CAGTATGTTCGGCTTCCCATTC 3’) were obtained from Harvard Primer Bank (B2M; NCBI accession #NM_009735; https://pga.mgh.harvard.edu/primerbank/).

### Experimental design and statistical analysis

Eighty-four Lis1 KO animals were given the 2 × 8 mg tamoxifen regimen; 100% of these animals began to exhibit neurologic symptoms (leg clasping, kyphosis, decreased motility) within a week. Of these, 18 died during that time, and 66 were killed when symptoms became severe; 38 no flLis1 control and 73 no Cre control mice were also given the 2 × 8 mg tamoxifen regimen. None of the controls showed any evidence of neurologic disorder or malaise, but most were killed at the same time as the Lis1 KO animals to compare results. However, six each of the control strains were monitored for four weeks after 2 × 8 mg tamoxifen and showed no symptoms. We also conducted mock injections of vehicle alone in 12 Lis1 KO animals. These controls also showed no symptoms.

Table 2 describes the statistical methods used for all quantified experiments. The *p* values were obtained using Excel or GraphPad Prism 5.

**Table 2.** Statistics used in the indicated experiments

Description	Data structure	Type of test	Statistical value
Axonal varicosities (Fig. 2G)	Non-normal	Mann–Whitney test	*p* < 0.0001
Retrograde transport (Fig. 2H)	Normal	*t* test	*t*(52) = 7.746 *p* = 3.2 × 10^−32^
Percentage of neurons with axons (Fig. 2I)	Normal	*t* test	*t*(7) = 3.2 *p* = 0.147
Axon length (Fig. 2I)	Normal	*t* test	*t*(190) = 5.0 *p* = 1.04 × 10^−6^
Nuclear enlargement index (Fig. 6D)	Normal	*t* test	*t*(912) = 19.55 *p* = 3.6 × 10^−73^
Centroid displacement index (Fig. 6F)	Non-normal	Mann–Whitney test	*p* < 0.0001
Lis1 mRNA in brainstem (Fig. 7B)	Normal	ANOVA	*F*(2, 19) = 5.033 *p* = 0.0176
Lis1 mRNA in heart (Fig. 7D)	Normal	ANOVA	*F*(2,16) = 19.065 *p* = 5.83 × 10^−5^

## Results

### Lis1 is expressed in adult mouse tissues, prominently in the nervous system

As detected by automated capillary immunoblotting, levels of Lis1 protein were only modestly lower in adult brain than embryonic brain protein samples (Fig. 1). Substantial Lis1 was also observed in adult spinal cord and dorsal root ganglia. While Lis1 could be detected in adult heart, liver, kidney, lung, and diaphragm, the levels were much lower in all non-nervous tissues tested than in brain, spinal cord and DRG. Therefore, Lis1 may function in other tissues, but is either present in fewer cells or at lower amounts per cell.

### KO of Lis1 by Cre-mediated recombination causes axon transport and growth defects in cultured adult sensory neurons

All mouse strains used in these studies are described in Table 1. To test the effectiveness of the Act-Cre-ER model for inducing recombination in neurons and reducing Lis1 expression, DRGs from Lis1 KO mice and the no Cre controls were dissociated and cells cultured for 24 h. Cultures were treated with 4-OHT for an additional 72 h. No tamoxifen-induced recombination was observed in the no Cre control cultures, as detected by prominent tdTomato fluorescence but no GFP (Fig. 2*A*). In contrast, substantial recombination was observed in the majority of neurons in the Lis1 KO cultures, detected by the presence of bright GFP fluorescence (Fig. 2*B*). GFP was observed in both axons and cell bodies. Also, Lis1 protein levels in extracts prepared from Lis1 KO cultures was greatly reduced relative to no Cre control cultures (Fig. 2*C*). Together these findings demonstrate effective KO of Lis1 by bath-applied 4-OHT in cultures of Lis1 KO mice but not of no Cre controls.

Intraperitoneal injection of tamoxifen into adult Lis1 KO mice also resulted in recombination in DRGs. For this experiment we injected 8 mg of tamoxifen on two consecutive days (2 × 8 mg regimen; see Materials and Methods) and harvested DRGs on day 4 after the first injection. Bright GFP fluorescence was observed in neuronal plasma membranes and satellite cells in DRG sections from these animals (Fig. 2*D*). No GFP was detected in DRGs from no Cre control animals injected at the same time (data not shown). Dissociated cultures prepared from no Cre control DRGs showed only tdTomato fluorescence (Fig. 2*E*), while Lis1 KO cultures exhibited bright GFP fluorescence indicative of substantial recombination (Fig. 2*F*). We first immunostained these cultures for NF to label axonal processes specifically so that images could be analyzed using automated software algorithms. However NF immunoreactivity was much more prominent in axon endings in Lis1 KO axons, possibly reflecting altered NF transport (Fig. 2*E*,*F*). Because of this we used the GFP and tdTomato signals to manually count varicosities. As predicted, the KO neurons had significantly more varicosities than the no Cre controls (Fig. 2*G*). Although varicosities occur normally at sites of growth cone pausing or sites of branch formation (Rees et al., 1976; Malkinson and Spira, 2010) an increased number is often associated with axonal blockages due to transport defects (Liu et al., 2012). Indeed, reduced retrograde transport of acidic organelles in living axons was observed in Lis1 KO axons compared to no Cre controls (Fig. 2*H*) consistent with other studies in adult rat DRG neurons where Lis1 was depleted using siRNA transfections (Pandey and Smith, 2011). We also observed that Lis1 KO axons appeared shorter. To quantify this we measured total axon length per neuron using a mixture of neuron-specific antibodies to ensure uniform labeling of axons (Fig. 2*I*). On average, Lis1 KO neurons had significantly shorter axons. There were also fewer neurons that had extended axons on day 2 after plating, indicating a delay in the onset of axon regeneration (Fig. 2*I*).

### Lis1 KO causes a severe phenotype in adult mice

**Figure 3.**
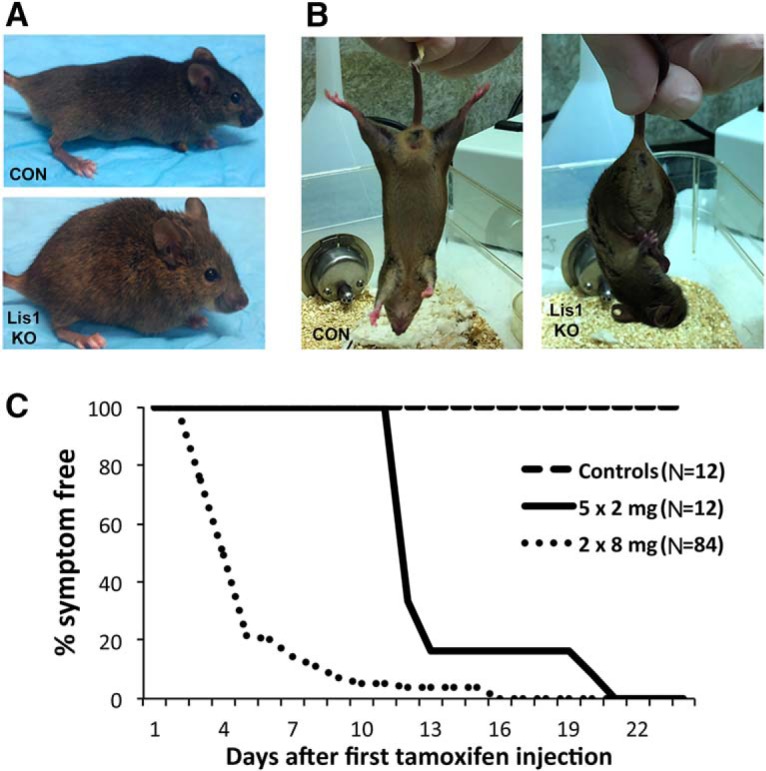
Lis1 KO via intraperitoneal tamoxifen injection in adult mice results in a severe phenotype. Lis1 KO mice exposed to tamoxifen invariably displayed spinal kyphosis (***A***, lower panel) and hind leg clasping (***B***, right panel). Neither was observed in control animals (CON) at any time. This includes the no Cre, no flLis1, Lis1 KO het, or mock-injected Lis1 KO animals. Both Lis1 KO and control mice were killed as soon as kyphosis and leg clasping became apparent in the KO animals. Phenotypes arose with latencies depending on the specific tamoxifen-dosing regimen (see Materials and Methods). ***C***, Symptom-free survival curves show that the latency is shorter for the 2 × 8 mg regimen (*N* = 84) compared to the 5 × 2 mg regimen (*N* = 12). Control mice were killed at the same time as the Lis1 KO mice for recombination and expression studies. However, six no Cre control mice and six no flLis1 control mice receiving the 2 × 8 mg dosing regimen survived symptom free for three weeks before they were killed (total *N* = 12).

Surprisingly, the 2 × 8 mg regimen caused a rapid decline in health of Lis1 KO animals, with spinal kyphosis (Fig. 3*A*) and hind leg clasping (Fig. 3*B*) observed 4 d after the first injection. None of the control animals showed this phenotype. In early experiments animals died within a week after the first injection, and mice were subsequently killed as soon as they began to exhibit symptoms, typically on days 3–5. Animals that were given a different regimen of tamoxifen, 2 mg injected for five consecutive days (5 × 2 mg regimen), remained non-symptomatic for nearly two weeks, after which they exhibited similar symptoms as observed in the 2 × 8 mg regimen. Figure 3*C* shows symptom-free survival duration plots for these animals. No differences were observed between male and female animals. Most animals were 2 months old at the time of injection, but similar responses were observed in older animals (4-5 months). Control animals did not exhibit any symptoms but were typically killed at the same time as KOs to be able to compare tissues for extent of recombination and Lis1 expression levels. However, six no Cre control and six *CAG-cre/Esr1* animals that received the 2 × 8 mg tamoxifen regimen lived for over a month with no detectable symptoms (Fig. 3*C*).

### Temporal differences in tamoxifen-induced recombination in distinct brain regions of Lis1 KO mice

**Figure 4.**
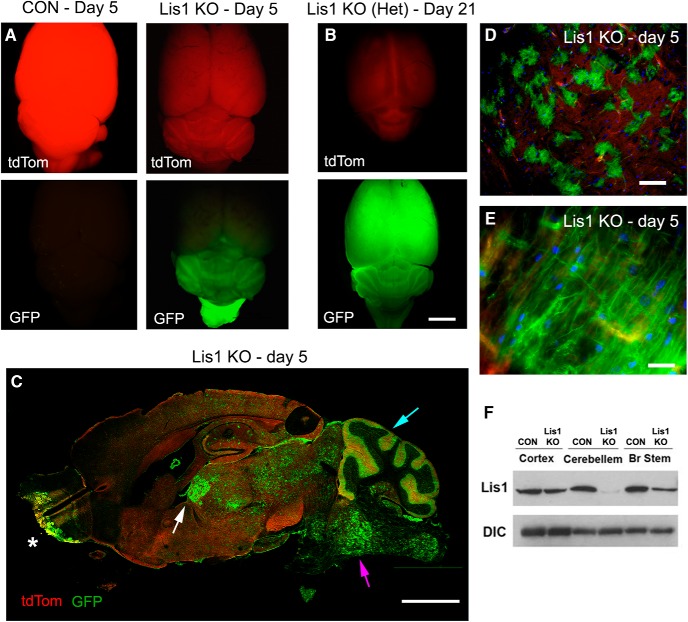
Cre-dependent recombination in the brain after tamoxifen injection. All data in this figure are representative of observations from a minimum of *N* = 4 animals of each genotype. ***A***, On day 5 after the 2 × 8 mg tamoxifen regimen, no Cre control brains (CON, day 5) had bright dtTomato fluorescence (top left panel), but no GFP fluorescence indicative of recombination (lower left panel). Lis1 KO mice (Lis1 KO, day 5) showed reduced dtTomato fluorescence (top right panel) and expressed EGFP primarily in the hindbrain, indicating that Cre activity was pronounced in this brain region (lower right panel). ***B***, Lis1 KO het mice [Lis1 KO (Het), day 21], which showed no sign of neurologic problems through day 21 after the injection had substantial GFP expression throughout the brain at that time. ***C***, A sagittal section of a Lis1 KO brain on day 5 (Lis1 KO, day 5) shows mosaic recombination in midbrain (white arrow), hindbrain (magenta arrow), and cerebellum (blue arrow), with widely scattered GFP-positive cells in cortex and hippocampus. Recombination also occurs in olfactory bulb (asterisk). ***D***, Using higher magnification, GFP-positive cells in the midbrain can be seen interspersed with cells that have not yet undergone recombination. ***E***, Fibers labeled with GFP are clearly visible in the brainstem. ***F***, Lis1 expression is reduced in extracts from brainstem and cerebellum of Lis1 KO mice compared to no Cre controls. Scale bars: 5 mm (***A–C***), 100 µm (***D***), and 20 µm (***E***).

Lis1 KO mice and no Cre control strains were given the 2 × 8 mg tamoxifen regimen. As expected, the no Cre control brains only exhibited tdTomato fluorescence on day 4 after the first injection (Fig. 4*A*, left). In contrast, bright GFP fluorescence was detected in Lis1 KO mice, but surprisingly, primarily in midbrain and hindbrain regions, with little fluorescence detected in the cortices (Fig. 4*A*, right). A similar result was observed in no flLis1 control so the recombination pattern is similar regardless of whether or not the mice carry the floxed Lis1 alleles.

Although the severity of the phenotype in Lis1 KO mice prevented examination of recombination at later times, no flLis1 control animals and Lis1 KO hets remained symptomless for at least three weeks after the 2 × 8 mg tamoxifen regimen. These animals showed substantial recombination observed across the entire brain, including the cortex, demonstrating variable rates of recombination in different brain regions with this 2 × 8 mg tamoxifen regimen (shown for Lis1 KO het in Fig. 4*B*).

In Lis1 KO mice recombination was observed throughout the medulla pons, midbrain and cerebellum and into the spinal cord, but only sparsely in the cortex and hippocampus (Fig. 4*C*). At this level of analysis, the most prominent recombination in the cerebellum occurs in the molecular layer in linear profiles reminiscent of Bergmann's glia. Substantial recombination was also observed in the olfactory bulb and choroid plexus (Fig. 4*C*). GFP-positive cells in the brainstem appeared stellate in shape (Fig. 4*D*). White matter tracks in the brainstem and cervical spinal cord were also GFP-positive (Fig. 4*E*). This GFP distribution correlates with the reduced Lis1 expression observed in brainstem and cerebellum but not in cortex (Fig. 4*F*).

### Lis1 KO occurs in both neurons and glia

**Figure 5.**
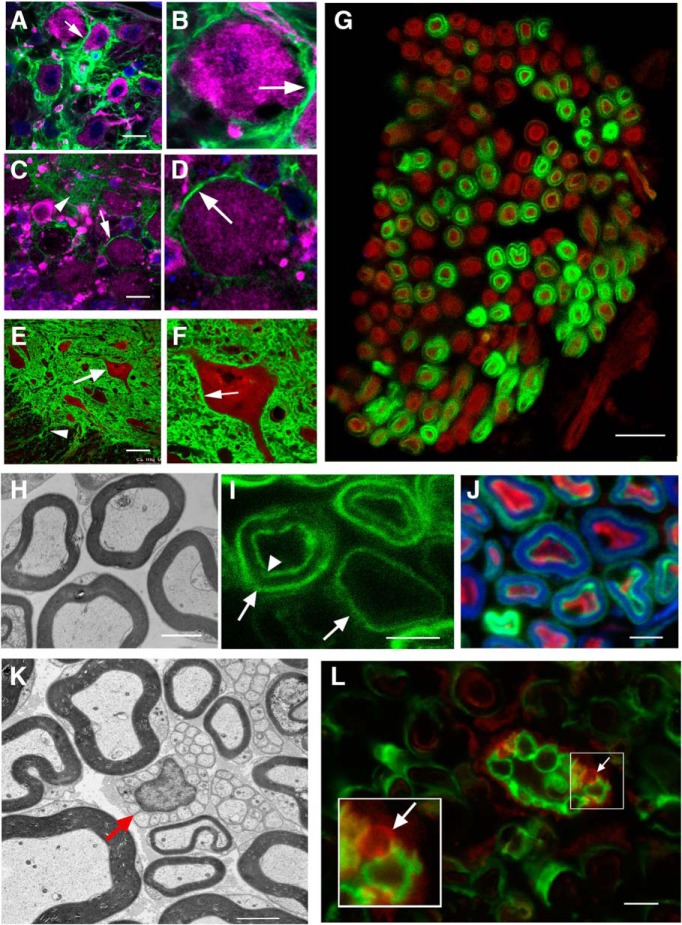
Both neurons and glia show evidence Cre-dependent recombination. All data in this figure are representative of observations from a minimum of *N* = 4 animals of each genotype. ***A***, GFP-positive neuropil surrounds MAP2-positive neurons (magenta) in a brainstem region thought to be the nucleus ambiguous in a mouse exposed to the 2 × 8 mg tamoxifen regimen. ***B***, The neuron indicated in ***A*** has been digitally enlarged to show details. The arrow points to possible neuronal plasma membrane. ***C***, Purkinje cells in the cerebellum stained with MAP2 (magenta) also have GFP-positive plasma membranes (white arrow) indicating that recombination occurred in these neurons. Neuropil in the molecular layer is also GFP-positive (arrowhead). ***D***, The neuron in C has been digitally enlarged to show detail. ***E***, GFP-positive neuropil surrounds a motor neuron (white arrow) labeled with ChAT (red) in the anterior horn of the thoracic spinal cord. GFP-positive fibers (arrowhead) can be seen coursing toward the ventral root. ***F***, The motor neuron indicated in E is digitally enlarged to show detail. The white arrow points to apparent neuronal plasma membrane. ***G***, A cross section through the phrenic nerve shows concentric rings of GFP around approximately half of the NF-positive axons (red). ***H***, EM of a cross section through a WT mouse nerve showing myelinated axons. ***I***, GFP can be observed as two concentric rings or single rings (arrows, outer ring, arrowhead, inner ring). ***J***, The area between concentric rings is positive for myelin basic protein (blue), while the inside of the inner ring is positive for NF (red). ***K***, EM of a cross section of WT mouse sciatic nerve showing Remak bundles of unmyelinated axons surrounded by a single glial cell (red arrow). ***L***, Cross section of sciatic nerve from Lis1 KO mouse with a Remak bundle containing some GFP-encircled axons, and some without encircling GFP (arrow, positive for tdTomato only). Inset is digitally enlarged to show an axon without recombination (red, arrow) alongside recombined axons (green). Scale bars: 10 µm (***A***, ***C***, ***G***), 30 µm (***E***), and 2 µm (***H–L***).

In cryosections of Lis1 KO DRGs (2 × 8 mg regimen), recombination was observed in both neurons and satellite cells (Fig. 2*C*). Membrane-targeted GFP was observed along neuronal membranes in the brainstem, Purkinje cells in the cerebellum, and motor neurons in the spinal cord (Fig. 5*A–F*). It was more difficult to distinguish neuronal processes from glia or axons from dendrites in the neuropil. Since Lis1 depleted DRGs neurons show altered axon transport and DRGs cultured from Lis1 KO animals showed signs of altered axon transport, we examined cross sections of phrenic, vagus, and sciatic nerves, as well as spinal cord ventral roots. Two concentric rings of GFP were typically observed around myelinated axons (Fig. 5*G–J*). The outer ring flanked the periphery of the myelin sheaths (Fig. 5*I*) that stained for myelin basic protein (Fig. 5*J*). GFP was not observed in the tightly packed myelin sheath itself, so the outer ring likely represents the plasma membrane of the myelinating glial cell. The inner ring was juxtaposed along the axoplasmic membrane of the ensheathed axon, and we interpret this as representing tamoxifen-induced recombination in axons of Lis1 KO neurons. This interpretation is strengthened by the observation that only an outer GFP ring was observed in some myelinated axons (Fig. 5*I*), which would be unlikely if the inner GFP-positive ring was also part of the myelinating Schwann cell. Unmyelinated C-fiber bundles in the sciatic nerve are ensheathed by membranes of Schwann cells that do not form myelin. These “Remak bundles” can be observed by EM (Fig. 5*K*). Red and green fluorescent rings were often observed in the same Remak bundles indicating that some, but not all axons in the Remak bundle had undergone recombination (Fig. 5*L*). Together these data support the *in vitro* finding that recombination, and by inference, Lis1 KO, occurs in both neurons and glia. The preponderance of recombination in the midbrain, hindbrain, and PNS, coupled with reduction in Lis1 protein levels in these regions, suggest that Lis1 KO in neurons and glia in these regions contributes to the observed neurologic phenotypes.

### Brainstem neurons show chromatolysis in Lis1 KO mice

**Figure 6.**
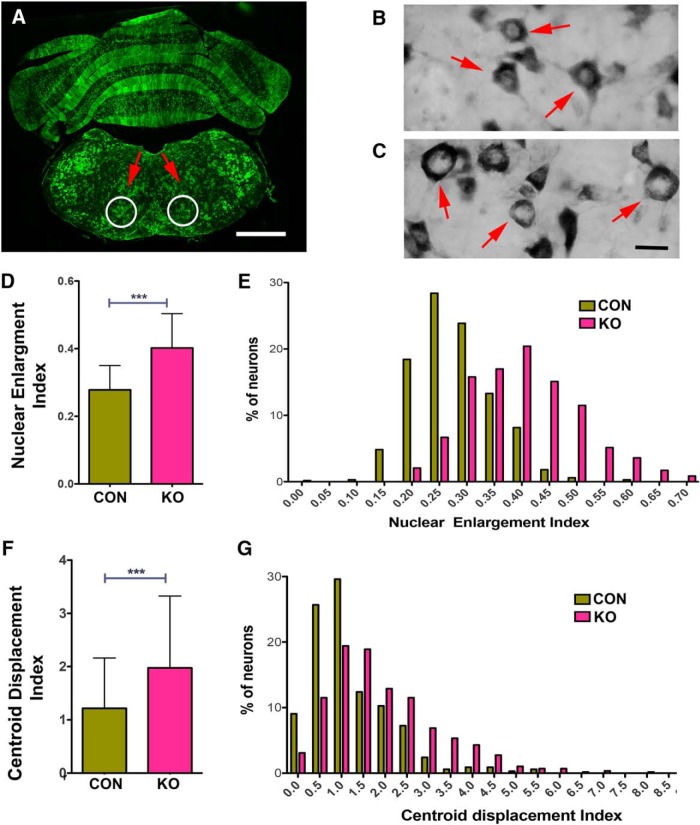
Brainstem neurons in Lis1 KO mice exhibit signs of chromatolysis. ***A***, A coronal section through the hindbrain on day 4 after the 2 × 8 mg tamoxifen regimen shows extensive recombination in the ventral brainstem containing cardiorespiratory centers. White circles indicate the region used in the analyses of chromatolysis. ***B***, ***C***, Sections were stained with toluidine blue to determine the size and position of the nucleus in neurons in the indicated regions. The neurons in B are from a no flLis1 control mouse. The neurons in C are from a Lis1 KO animal. ***D***, A nuclear enlargement index (see Materials and Methods) was used to compare nuclear enlargement in no flLis1 controls (CON) and Lis1 KO (KO). ***E***, The histogram shows the distribution of this index in CON and Lis1 KO neurons. ***F***, The position of the nucleus within the soma was also determined using the centroid displacement index (see Materials and Methods). This involves determining the centroid position of both the nucleus and soma and calculating the total displacement distance (µm) of the nuclear centroid from the somal centroid. ***G***, Histogram showing the distribution of CDI found in CON and KO neurons. Bars indicate mean ± SD. Brainstem sections from three no flLis1 control and four Lis1 KO mice were used in the chromatolysis study. This includes analysis of 331 control neurons and 583 Lis1 KO neurons. Significance determined by Student’s *t* test (***D***), or Mann–Whitney test (***F***); ***p < 0.001 (see Table 2 for details). Scale bars: 1 mm (***A***) and 10 µm (***B***, ***C***).

Figure 6*A* shows GFP expression in a coronal section through the hindbrain, with a dense concentration of GFP-positive cells in the ventral hindbrain. This area contains nuclei that are vital for cardiorespiratory function, and complete functional loss of these neurons would result in rapid death (Melov et al., 1998; Quintana et al., 2012). Impairment of axonal transport or other dynein-dependent processes in this region could account for the severe phenotype in Lis1 KO animals. Experimental axotomy and diseases that involve axonal dysfunction can produce the cell body response of chromatolysis (Cragg, 1970; Hanz and Fainzilber, 2006). Though the mechanisms underlying chromatolysis remain hypothetical, the process is characterized by nuclear swelling and nuclear acentricity, both of which were observed in GFP-rich regions in coronal sections through the brainstem of Lis1 KO mice (Fig. 6*C*). The nuclei were significantly larger and more acentric than controls providing evidence for axonal dysfunction in these neurons (Fig. 6*D–G*).

### Lis1 loss in the hindbrain is the most likely cause of the KO phenotype

**Figure 7.**
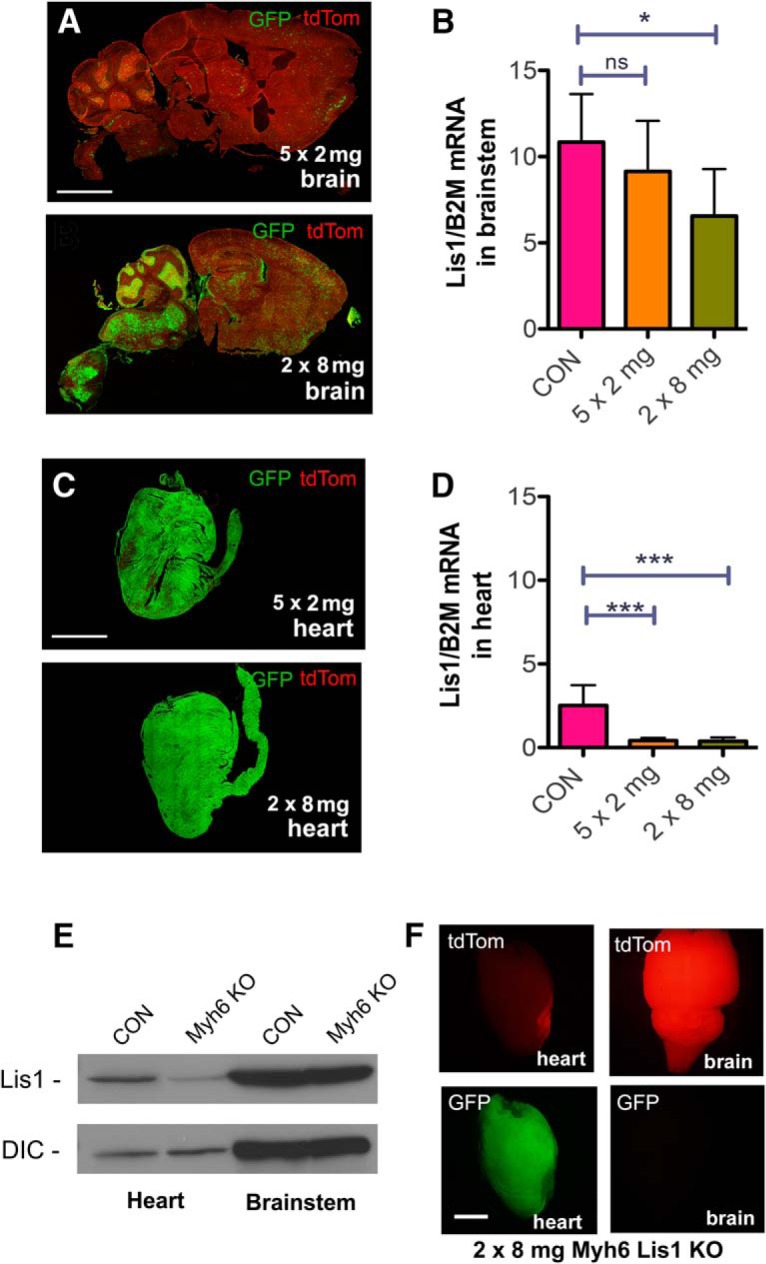
Comparing the effect of Lis1 KO in brainstem and heart. ***A***, Sagittal brain sections of Lis1 KO mice 5 d after the initial injection of either five injections of 2 mg tamoxifen (top) or two injections of 8 mg tamoxifen (bottom). The 2 × 8 mg treatment resulted in much higher GFP expression than the 5 × 2 mg treatment, particularly in the brainstem and cerebellum. ***B***, Lis1 mRNA levels normalized to B2M mRNA levels from brainstem of no Cre control mice injected with 2 × 8 mg tamoxifen (CON), and Lis1 KO mice injected with either 5 × 2 or 2 × 8 mg tamoxifen. Lis1 mRNA levels were significantly decreased in brainstem of 2 × 8 mg animals, but not 5 × 2 mg animals, relative to no Cre controls, 5 d after initial injection. ***C***, Sections of heart from 5 × 2 mg (top)- and 2 × 8 mg (bottom)-treated Lis1 KO mice. Both the 2 × 8 and 5 × 2 mg treatments resulted in similar levels of GFP expression in heart. ***D***, Lis1 mRNA levels normalized to B2M mRNA levels from heart of 2 × 8 mg-injected no Cre control (CON)-, 5 × 2 mg-, and 2 × 8 mg-treated mice. Lis1 mRNA levels were reduced significantly in both the 5 × 2 mg- and 2 × 8 mg-treated mice relative to the no Cre control but were not significantly different from each other. ***E***, Western blotting of brainstem and heart lysates from cardiomyocyte-specific Myh6 KO mice show reduced levels of Lis1 protein in heart, but not brainstem compared to no Cre control mice (CON). Dynein intermediate chain (DIC) was used as a loading control. ***F***, Whole mount brain (right) and heart (left) from Myh6 KO mouse show recombination (GFP) in heart but not brain. Data in ***A***, ***C***, ***E***, ***F*** are representative images from *N* = 3 mice for each genotype. The RNA quantification in ***B***, ***D*** represent mean of data from *N* = 3 animals of each treatment and genotype ± SD. Significance in ***B***, ***D*** determined by one-way ANOVA; *p < 0.05, ***p < 0.001 (see Table 2 for details). Scale bars: 5 mm (***A***, ***C***) and 2 mm (***F***).

Lis1 KO mice receiving the 2 × 8 mg tamoxifen regimen had a much more rapid onset of symptoms compared to the 5 × 2 mg regimen (Fig. 3*C*). Indeed, at a time when the 2 × 8 mg mice were severely affected (day 5), the 5 × 2 mg animals had no overt symptoms. Substantially more recombination was observed in the brains of 2 × 8 mg animals compared to 5 × 2 mg animals, which correlates with the onset of severe symptoms (Fig. 7*A*) and the level of Lis1 mRNA in the brainstem (Fig. 7*B*). In contrast, recombination in the heart was similar in both sets of mice (Fig. 7*C*), as were Lis1 mRNA levels, which were reduced equally with both regimens (Fig. 7*D*). This indicates that Lis1 KO in the heart is less likely to be responsible for the early onset of symptoms. Other tissues with sporadic GFP (lung, liver, and kidney) also showed a similar degree of recombination with both regimens, supporting the idea that the hindbrain loss of Lis1 contributes significantly to the KO phenotype.

We performed another experiment to directly test contributions of Lis1 KO in the heart to the phenotype observed in Lis1 KO mice by generating an inducible KO in which Cre-ER is driven by a Myh6 promotor (Myh6 KO; Sohal et al., 2001). As expected, tamoxifen injections (2 × 8 mg) reduced Lis1 expression in the heart but not in the brainstem of these mice (Fig. 7*E*). Moreover, robust GFP expression, and thus Myh6 KO-dependent recombination, was observed in the heart, but not in the brain (Fig. 7*F*). Despite significant recombination in the heart, 0/12 mice showed any detectable phenotype, and all lived apparently symptom free, until killed four weeks later. Together these data provide evidence that loss of Lis1 in midbrain/hindbrain neurons is responsible for severe phenotype in Lis1 KO mice.

## Discussion

We show that Lis1 KO by tamoxifen-induced recombination causes neuropathology and lethality in adult mice. The finding that Lis1 continues to play a vital role after the majority of mitosis and migration in the brain has occurred supports the idea that Lis1 regulates additional cellular processes such as axonal transport. Given the preponderance of evidence that Lis1 regulates cytoplasmic dynein coupled with the observed temporal and spatial pattern of Cre-mediated recombination, the simplest explanation for the severe phenotype caused by Lis1 depletion is that defective axon transport results in pathologic changes in midbrain, hindbrain, spinal cord, and DRG neurons. That these areas are critical to the phenotype is supported by the correlation between the location and extent of recombination (and thus Lis1 loss), and the onset of symptoms using different tamoxifen dosing regimens. At the onset of severe neurologic symptoms (∼4–5 d) the 2 × 8 mg tamoxifen regimen produced substantial recombination in cells in those regions. At the same time point in animals exposed to the 5 × 2 mg tamoxifen regimen no symptoms were present and much less recombination was observed in these areas. Unlike the nervous system, other tissues (heart, lung, liver, kidney) had a similar degree of recombination using both regimens, arguing for an important nervous system contribution to the Lis1 KO phenotype. This is further supported by evidence of chromatolytic neurons in the brainstem and axon transport defects and varicosities in DRG neurons cultured from Lis1 KO mice. Axons of the vagus, phrenic, sciatic nerves and ventral roots contained the Cre reporter (GFP) indicating that recombination had occurred in neurons. Lis1 depletion in neurons with axons running in those nerves could contribute to the Lis1 KO phenotype. For example the vagus nerve contains autonomic axons emerging or converging on the nucleus ambiguous, solitary nucleus, dorsal nucleus of vagus, and spinal trigeminal nucleus, and is critical for cardiorespiratory control. The phrenic nerve contains sympathetic, sensory and motor axons innervating the diaphragm, mediastinal pleura, and pericardium. Brainstem perturbations in animals can lead to death due to cardiorespiratory disruption (Talman and Lin, 2013; Piroli et al., 2016; Sun et al., 2017). Thus, the idea that Lis1 KO in brainstem neurons is a lethal event in mice is not too farfetched.

Sensory and motor neurons with axons in the sciatic nerve are less likely to contribute to the death of the animals, but may contribute to the leg clasping and kyphosis observed in 100% of Lis1 KO mice. Also, the finding that axon growth by DRG neurons in culture is compromised by Lis1 depletion indicates that peripheral nerve regeneration *in vivo* could be affected by changes in Lis1 expression. The role of microtubule motors in developmental axon growth and axonal regeneration after injury is complex. In some studies, motor activity appears to be a negative regulator of growth. Retrograde target derived signals prohibit growth in mature connected neurons (Smith and Skene, 1997). Transport-dependent length-sensing signals have an inverse relationship with axon length, in those studies dynein or kinesin knockdown or a specific dynein mutation caused adult DRG axons to be longer (Kam et al., 2009; Rishal et al., 2012). Finally, stimulation of dynein by a BICD2 mutation resulted in shorter axons in cultured hippocampal neurons (Huynh and Vale, 2017).

On the other hand, there are also many studies that indicate that both anterograde and retrograde signals promote regenerative growth (Mar et al., 2014; Kalinski et al., 2015). For example, a pro-growth injury signal that is required for axonal regeneration in the sciatic nerve requires retrograde transport by dynein (Ben-Yaakov et al., 2012). Dynein can push the cytoskeleton forward during axon initiation and elongation in developing axons (Dehmelt et al., 2006; Roossien et al., 2014), and DHC knockdown reduced microtubule movements into growing axons and stunted axon outgrowth in cultured adult PNS neurons (Ahmad et al., 2006). While our data focused on regeneration of adult axons, others have also found that depletion of Lis1 leads to decreased developmental axon growth (Grabham et al., 2007; Kumamoto et al., 2017). Taken together, these data suggest that Lis1 knockdown could reduce transport of regenerative signals and also disrupt dynein-dependent cytoskeletal changes required for axon growth. Because Lis1 overexpression stimulated dynein-dependent organelle transport (Pandey and Smith, 2011), it will be interesting to determine whether Lis1 overexpression also negatively impacts axon growth.

While it is critical to know the organs, tissues and cells that contribute to the severe Lis1 KO phenotype, it is equally important to determine which are less affected by Lis1 depletion. With respect to the midbrain/hindbrain, spinal cord, and DRG neurons, we cannot state that some neuronal subtypes are more affected by Lis1 KO than others, and in fact, suspect that disruption in all of them could be a contributing factor to the phenotype, as suggested above. With respect to other brain regions, including the cerebral cortex, hippocampus, striatum, pallidum, and hypothalamus, *Lis1* KO animals died before significant recombination occurred in these regions. However, we expect that Lis1 is important in all axons; signs of pathology may have become apparent in these regions had Lis1 KO animals survived long enough for recombination to occur. Our heterozygous *Lis1* KO animals exhibited substantial recombination in all brain regions by three weeks after 2 × 8 mg tamoxifen injection but did not exhibit leg clasping, kyphosis or lethality. It remains to be determined whether signs of axonal dysfunction will arise in cortical regions of heterozygous Lis1 KO mice as animals age. Another group used a similar inducible system to examine hippocampal function following heterozygous *Lis1* KO in six-week-old mice (Hunt et al., 2012). They found an increase in excitatory synaptic input to granule cells in the absence of neuronal positioning defects. The molecular and cellular underpinnings of this are not known, but our data suggest that it might involve axonal transport disruption. In our study, a much more limited Lis1 depletion was accomplished by stereotaxic injections of 4-OHT into lateral ventricles. This did not produce any obvious symptoms (leg clasping, kyphosis or death) probably because recombination had occurred very sparsely and mainly in glial cells (data not shown). Ultimately, more selective examination of the role of Lis1 in adult neuronal circuits will require using Cre-driver(s) specific for different neuronal populations. Some glial cells exhibited Cre-mediated recombination in our Lis1 KO mice. Glial cells in cortical, hippocampal and DRG cultures express Lis1 (Smith et al., 2000), and there was substantial recombination in Bergmann’s glia, astrocytes and Schwann cells, so depletion of Lis1 in any of these cells could theoretically contribute to Lis1 KO phenotypes. Glial specific Lis1 KO may allow us to answer this question.

With respect to other tissues in the mouse, we can state unequivocally that the severe Lis1 KO phenotype was not due to Lis1 depletion in cardiomyocytes. Depletion in liver, lung, and kidney was likely highly mosaic at the time when symptoms were severe because recombination was sparse in these tissues, and was similar on day 5 using both tamoxifen regimens. Urine and blood was tested for changes that might signal kidney, bladder, liver or intestine defects, but no significant changes were found (data not shown). Thus, while our data suggest that Lis1 depletion in these tissues does not produce the rapidly lethal phenotype, to fully answer this question, we will need to use organ or cell specific Cre-ER drivers.

While our studies unequivocally demonstrate a vital role for Lis1 in adult mice and strongly support a role in axon transport, Lis1 and dynein both function in dendrites, at least during development. For example, dynein-based transport occurs in dendrites in rodent hippocampal cultures (Kapitein et al., 2010; Ayloo et al., 2017), and signs of transport defects are observed in motor neurons in the adult *Loa* dynein mutant mouse (Wiggins et al., 2012). Interestingly, Lis1 knockdown by shRNA expression disrupted the translocation of excitatory synapses in developing interneuron dendrites in hippocampal cultures and organotypic slices (Kawabata et al., 2012). Moreover, two-photon microscopy showed altered spine morphology dynamics in three-week-old Lis1^±^ mice (Sudarov et al., 2013). Together, these reports suggest that Lis1 dysfunction in dendrites in the midbrain, hindbrain, and spinal cord could contribute to the Lis1 KO phenotypes.

Uncovering a role for Lis1 in axonal or dendritic transport in post-mitotic, connected neurons is interesting for several reasons. First, if axonal transport is compromised in LIS, it could contribute to the seizures (which become increasingly frequent and severe) and the early lethality typical of the disorder. Axon transport defects, unlike defects that arise because of brain malformations that occur *in utero*, might be ameliorated with drugs that target the dynein regulatory machinery. Second, dynein-related proteins are linked to many neurodegenerative diseases (Rees et al., 1976; Wider and Wszolek, 2008; Weedon et al., 2011; Harms et al., 2012; Neveling et al., 2013; Oates et al., 2013; Peeters et al., 2013). In fact, respiratory problems in the late-onset disorder, Perry Syndrome, are the cause of lethality in humans (Wider and Wszolek, 2008). To treat such disorders, it is critical to understand the mechanisms regulating dynein in axons. In most cell culture studies, Lis1 overexpression stimulated, and Lis1 disruption reduced processivity (Liu et al., 2000; Smith et al., 2000; Pandey and Smith, 2011; Shao et al., 2013; Klinman and Holzbaur, 2015; Villarin et al., 2016). However, one transport study indicated that Lis1 knockdown increased mitochondrial transport (Vagnoni et al., 2016), and several *in vitro* biophysical studies showed that Lis1 inhibited processivity of purified dynein (Yamada et al., 2008; McKenney et al., 2010; Huang et al., 2012). More recent assays using purified proteins are beginning to reveal how this might occur at the molecular level in the context of other dynein regulators like dynactin and BICD2 (Baumbach et al., 2017; DeSantis et al., 2017; Gutierrez et al., 2017). In those studies, Lis1 dramatically increased dynein processivity. Interestingly BICD2 mutations that cause SMALED stimulate dynein processivity, so motor activity must be finely tuned (Huynh and Vale, 2017). Kinase pathways that impact dynein function have been identified. CDK5, mutations in which have been linked to LIS (Magen et al., 2015; Parrini et al., 2016) and other kinases phosphorylate and regulate the Lis1- and dynein-interacting protein Ndel1 (Hebbar et al., 2008; Pandey and Smith, 2011). Phosphorylation also regulates motors directly (Gibbs et al. 2015). We recently reported that insulin-dependent inhibition of GSK3β, a kinase with a growing list of neurologic disease links (Dell'Osso et al., 2016), phosphorylates dynein and regulates its interactions with Ndel1 and APC (Gao et al., 2015; Gao et al., 2017). It will be interesting to determine whether these pathways can be manipulated to alter the severity of the Lis1 KO phenotype, and if they can be used in trying to alleviate symptoms of patients with diseases caused by transport defects.

## Synthesis

Reviewing Editor: Orly Reiner, Weizmann Institute of Science Decisions are customarily a result of the Reviewing Editor and the peer reviewers coming together and discussing their recommendations until a consensus is reached. When revisions are invited, a fact-based synthesis statement explaining their decision and outlining what is needed to prepare a revision will be listed below. The following reviewer(s) agreed to reveal their identity: Stephen King, Peter Baas

The eN-NWR-0350-17 manuscript describes an inducible Lis1 KO mouse. There have been conflicting models for Lis1 function developmentally and/or in adult animals. The approach taken here provides clear and overwhelming evidence that Lis1 has an essential role in adults. Within a week of KO induction, animals became severely ill and died. Analyses of Lis1 function in adults focused on brain tissue and found several interesting defects in the hind and midbrain, but not in the cortex, as might be expected based on lissencephaly phenotypes. Axons of affected cells showed transport defects along with increased numbers of varicosities. Both of these effects suggest that a subset of neurons may be more sensitive to Lis1-dependent dynein based axonal transport.

Overall, the paper was well written, with clear data presentation and the conclusions presented were generally supported by the data.

A few minor points need to be addressed for publication:

On page 14 the authors mention that Lis1 KO neurons have shorter axons, but the data is not shown, That is a very interesting point, and I would like to see that data in the manuscript.

On page 23 Figure 2G and H as well as in several other bar graph figures, the bars are described as being displayed mean +/- 95%. Acceptable norms in motility data is to show standard deviations and those should be included in all such bar graphs.

It was difficult to find the sample size for many experiments, as those numbers were in the “Culture analyses” methods section as opposed to in the sections where the tests were actually performed. This reviewer would like to see sample size (both N and n) presented along with the experimental methodologies whenever possible.

Please include an explicit and detailed paragraph about the organs, tissues or cells (especially in the nervous system) that seem less affected by Lis1 depletion. This is equally important as those that show a strong or moderate effect.

## Author Response

Rebuttal Letter

Please accept this revised submission of our manuscript entitled: “An essential post-developmental role for Lis1 in mice”. We were very pleased by the reviews and have addressed all of the concerns outlined in the synthesis.

1: It was requested that we provide data concerning an interesting observation that axons in DRG cultures from Lis1 KO mice appeared shorter. We have carried out more studies and provide careful quantification of this observation. The data indicate that fewer Lis1 KO neurons extend axons, and that those axons are significantly shorter. Thus reduced Lis1 expression, and by inference, defective dynein-dependent transport, is detrimental to axonal growth by cultured adult sensory neurons. This new data has been added as figure 2I, and new text, in red font, in the results, discussion and figure legend.

2. All bar graphs are presented with standard deviations rather than 95% confidence intervals.

3. Sample sizes for experiments (N and n) are now included in both the figure legends and in the methods sections relevant to those experiments. We also have included sample sizes and statistics used in the last section of Methods, called “Experimental Design and Statistical Methods”. The sample size information is present in red font.

4. A paragraph detailing which tissues were not affected by Lis1 KO was requested. We have included two paragraphs (red font) in the discussion. Some of this was reorganized from the previous discussion and some is new. We feel it adequately addresses this concern to the degree that the data allows us to address it.

We have uploaded each figure as a Tiff file, and look forward to seeing this work published in eNeuro.
